# Hospital Saturday and Street Collections

**Published:** 1893-10-21

**Authors:** 


### Hospital Saturday and Street Collections.

The recent frauds in connection with the Metropolitan Hospital Saturday Fund, and the discussion which took place at the meeting of the council on Saturday week, prove the faultiness of the system on which the street collections have heretofore been conducted in London. With a view of showing a better way of managing these collections, although, as we stated last week, we are of opinion that it is desirable to abolish all street collections of every kind, we have asked Mr. W. T. Smedley, the Honorary Secretary of the Birmingham Hospital Saturday Fund, which has been the most successful of all, to describe the Birmingham system. It will be seen that such frauds as those which have occurred in London could not be perpetrated under this excellent system. Mr. Smedley writes: The following is the method upon which the street collection is conducted in Birmingham: Each stand is under the superintendence of a lady known to members of the Executive Committee. Large boxes are provided with flaps on two sides, which serve the purpose of a table. These are delivered to the respective stands at six o'clock on the morning of the collection by vans. Each box has a drawer into which the money falls from the slit at the top, the key of which is retained at the office. In each box are three large canvas bags with the number of the box printed on. The town is divided into districts, two members of the Executive Committee being appointed to each as collectors. The whole of the collectors start from the office at two o'clock, six o'clock, and eight o'clock. They have a list of the stands, the number of the boxes at each, and the keys of the drawers. They go round, open the boxes, put the money from the drawer into one of the bags which they find inside, fasten it up in the presence of the lady having the management of the stall. Having gone their round, depositing the canvas bags in a large leather one, they return to the office with them. We have there about twelve or fourteen clerks acting as counters, two or more being allotted to each division. The members of the Executive Committee who have been out collecting bring their bags to these counters, and the contents of the bags are then counted, and when the collectors go on their next round they take to each lady a bill to put up showing the amount received at the stall up to the previous collection. I send you one of these bills herewith. This course is followed out on each round of collection, with the addition that on the last round a van follows the collectors and picks up the boxes as they are emptied, and returns them to the office the same evening. The whole of the money is counted and balanced before the counters leave the office, generally about two or three o'clock on Sunday morning, and a full list of the amounts taken at each stall appears in the papers of the Monday following. In fact, the bulk of the money is paid into the bank before it closes at nine o'clock on the Saturday evening. Taking the last two years as an example, the following were the figures paid on Saturday and Monday respectively: Saturday, 1892, £372 10s.; Saturday, 1893, £339 1s. 5d.; Monday, 1892, £72 13s. 4d.; Monday, 1893, £97 13s. 11d.

We do not look upon the street collection here so much as a source of income as a method of emphasising the fact that it *is* Hospital Saturday. You will doubtless remember that we only receive money on the one day, and for a few days following. The street collection is most valuable to us as a means of letting everybody know that it *is* Hospital Saturday, for I think the ladies make that fact very apparent.

